# Proteomic Identification of Differentially Expressed Proteins between Male and Female Plants in *Pistacia chinensis*

**DOI:** 10.1371/journal.pone.0064276

**Published:** 2013-05-17

**Authors:** Erhui Xiong, Xiaolin Wu, Jiang Shi, Xiaoyan Wang, Wei Wang

**Affiliations:** 1 Key Laboratory of Physiological Ecology and Genetic Improvement of Food Crops in Henan Province, Department of Plant Science, College of Life Science, Henan Agricultural University, Zhengzhou, China; 2 Department of Botany, College of Agronomy, Henan University of Science and Technology, Luoyang, China; University of Nottingham, United Kingdom

## Abstract

*Pistacia chinensis* is a strict dioecious plant with male and female flowers in individuals. In China, *P. chinensis* is widely planted for biodiesel oil due to high oil content in seeds. In practice it requires to grow more female plants for biodiesel production. At present, there are still no reliable methods for sex determination during the long juvenile stage of this species. In order to develop protein molecular markers for sex determination in *P. chinensis*, proteomic approach was used to identify differentially expressed proteins between male and female plants. Vegetative organs (leaf and stem) rather than reproductive organs/tissues were used for protein extraction so as to develop protein markers which can be used in siblings before flowering. Protein was extracted using a phenol-based protocol. By using two-dimensional electrophoresis, a total of 10 protein spots were found to be differentially expressed in leaf and stem between both sexes, of which 7 were successfully identified by mass spectrometry and matched to 6 functional proteins such as NB-ARC domain containing protein, light harvesting chlorophyll a/b binding protein, asorbate peroxidase (APX), eukaryotic translation initiation factor 5A2, temperature-induced lipocalin (TIL) and phosphoglycerate kinase (PGK). The sex-related difference displayed in a tissue-specific way, especially in stem. PGK existed in high abundance in stem phloem in the female, but was almost not detected in the male; APX and two TIL species were highly abundant in the stem of male plants, while their abundance was much lower in female plants. Moreover, these abundance differences were further confirmed in individual plants. Hence, it is assumed that APX, PGK and TIL might be promising candidates to serve as protein molecular markers for sex determination in *P. chinensis*. Our results form the basis for a further understanding of the biochemical mechanisms of sex determination in *P. chinensis*.

## Introduction

The great majority of flowering plants are hermaphrodite, having flowers with both male and female parts. On the other hand, less than 4% of plant species are dioecious, having male and female flowers in individuals [1]. Only a small fraction of dioecious plants have evolved sex chromosomes, such as X∶Y system in white campion (*Silene latifolia*), X∶A system in hop (*Humulus lupulus*) [2].

Most dioecious plants do not exhibit discernible sexual dimorphism before sexual maturity. A reliable method for sex determination in dioecious plants cultivated for fruit or seed at juvenile stage would greatly benefit breeding program. For this purpose, a range of sex-linked molecular markers such as RAPD, RFLP, AFLP and microsatellite have been generated [3]. In *Pistacia* species, male- or female-specific RAPD markers have been identified [4]–[6]. In *S. latifolia*, a gene encoding a male-specific protein is linked to the X chromosome [1]. Recently, a female-specific DNA marker has been discovered in the moss *Pseudocalliergon trifarium*, allowing a reliable determination of gender and sex ratio [7]. However, primary sex determining genes have not yet been identified in any dioecious plant species to date.

Chinese pistache tree (*Pistacia chinensis* Bunge), native to East Asia (especially China), is a strict dioecious species in the cashew family. It is a popular choice for street tree in urban settings [8]. In China, *P. chinensis* is widely planted for biodiesel oil because of high seed oil content (35–50%) [9]. Thus, female plants of *P. chinensis* have higher economic values than male plants. In practice it is required to grow more female plants for biodiesel production. Nursery-grown *P. chinensis* plants first flower at 6–10 year-age and at present there are no reliable methods for sex determination in this plant species prior to flowering. In addition, sex determination mechanisms in *P. chinensis* are still unknown.

As a powerful tool for protein identification and functional characterization, proteomics techniques have been widely used in plant biology. A proteomic analysis usually employs two-dimensional electrophoresis (2-DE) or multidimensional chromatography for protein separation and mass spectrometry (MS) for protein identification [10]. 2-DE-based proteome analysis is especially suitable for paired comparison of samples. In *P. chinensis*, male and female plants strictly maintain their respective sexual phenotypes [11] and an approximately 1∶1 sex ratio [12], which suggests the presence of a clear genetic basis of sex differences. Hence, it is expected that sex difference in this dioecious species should display at the proteome level.

The aim of this study was to find out differentially expressed proteins which could be related to sex difference in *P. chinensis*. Vegetative organs (leaf and stem), rather than reproductive organs/tissues, were used for protein extraction so as to develop protein markers which can be used in siblings before flowering. Leaf, stem xylem and stem phloem from both sexes were used for comparative proteomic analysis using 2-DE followed by MALDI-TOF MS/MS. Our results revealed significant and reproducible differences in protein profiles between male and female in *P. chinensis*.

## Materials and Methods

### Plant material

*Pistacia chinensis* is a deciduous, wind-pollinated, large shrub or small tree. There are no discernible morphological features between male and female, until adult plants (6–10 year-old) flower in spring when their sex can be easily distinguished by appearance of flowers. Male panicles (Figure S1, A) are pale green and female panicles (Figure S1, B) are purple.

Male and female wild plants of *P. chinensis* (approximately 10 to 40 years of age) were used for this study. The age of each individual was estimated by diameter at breast height, as described previously [13]. Leaves were sampled in summer, and twigs were sampled in automn and winter. Samples were manually selected, frozen immediately in liquid N_2_ and stored at −80°C until further analysis. No specific permits were required for the described field studies, which did not involve endangered or protected species.

### Preparation of acetone tissue powder

Leaf, stem xylem and stem phloem, rather than reproductive organs/tissues, were used for materials, because differentially expressed proteins in reproductive organs/tissues may not be suitable for sex determination of siblings at juvenile stage. One pair of healthy leaflets each compound leaf (Figure S1, C) were selected for preparation of acetone tissue powder. Frozen twigs were soaked in water for 30–60 min so as to facilitate the separation of xylem with phloem. Xylem and phloem were manually separated out. The phloem was pale green, indicating the presence of chlorophyll (Figure S1, D). Xylem exists inside the twigs and is less affected by the external conditions than leaf and stem phloem. It can be sampled all the year around, while leaves are only available during the growth season. In addition, xylem is more easily separated out from the twigs than phloem. Therefore, xylem, if appropriate, would be a good choice as material for sex determination.

Plant material was pulverized to a fine powder in liquid N_2_ using a mortar and pestle. The sample was transferred to a centrifuge tube and cold 10% trichloroacetic acid in acetone with 0.1 M dithiothreitol (DTT) was added. Samples were kept at −20°C for at least 2 h, and then centrifuged at 15000 g for 15 min at 4°C. The resulting pellet was washed 3 times by suspending in cold acetone and centrifuged as above between each wash. The pellet was air-dried and used for protein extraction.

### Protein extraction for two-dimensional electrophoresis

In order to reduce sampling variation, a mixed tissue powder (‘average sample’) from three different male or female individuals in each age group was used for protein extraction. For each independent experiment, samples from 12 plants (3 each sex in 2 age groups) were used. Protein was also extracted from individuals for paired comparative analysis. Protein extraction was performed in three biological replicates.

Protein was extracted using the phenol-based protocol [14], [15]. Tissue powder was homogenized in the extraction buffer (0.1 M Tris-HCl, pH 8.8, 2% SDS and 0.1 M DTT, 1∶10 g/ml) in a mortar. The extract was transferred into a fresh tube and then clarified by centrifugation at 15000 g for 10 min. The supernatant was mixed with equal volume of buffered phenol (pH 8.0, Sigma, P4557). Phases were separated by centrifugation at 15000 g for 5 min. The phenol phase was precipitated with 5 volumes of cold methanol containing 0.1 M ammonium acetate overnight (−20°C). Protein was recovered by centrifugation and washed twice with cold acetone. Air-dried protein pellet was dissolved in 2-DE rehydration buffer (7 M urea, 2 M thiourea, 4% CHAPS, 2% IPG buffer, 20 mM DTT and a trace amount of bromophenol blue). Protein extract was clarified by centrifugation prior to 2-DE. Protein was quantified by the Bio-Rad protein assay with bovine serum albumin as a standard.

### Two-dimensional gel electrophoresis

Protein (approximately 500 µg in 220 µl buffer) was loaded on 11 cm Immobiline gels (Bio-Rad) via passive rehydration overnight. After rehydration, proteins were separated by isoelectric focusing (IEF) using the PROTEAN IEF CELL system (Bio-Rad), starting with a low initial voltage of 300 V, then stepwise increases to 8000 V for a total of about 60 kVh. After IEF, gel strips were equilibrated for 15 min in the equilibration buffer containing 0.1 M Tris-HCl, pH 8.8, 2% SDS, 6 M urea, 30% glycerol and 0.1 M DTT, followed by another 15 min in another equilibration buffer in which 0.1 M DTT was replaced by 0.25 M iodoacetamide. Afterwards, proteins were resolved by SDS-PAGE using 12.5% polyacrylamide gels. Gels were stained in 0.1% CBB R-250 overnight and destained in 10% acetic acid. This study aimed to find out the most significant changes in protein profiles between male and female plants, therefore, relatively insensitive CBB staining was used to visualize proteins.

### Gel imaging and data analysis

Gel images were analyzed by PDQUEST (version 7.0, Bio-Rad) for spot detection, gel matching and statistical analysis of spots. The separate analysis for each male or female sample included alignment of gels to a reference image. Quantitative analyses were carried out after normalizing the spot volumes in all gels, in order to compensate for abundance related variations. Differential variations in spot abundance between male and female were tested with one-way ANOVA (p<0.05). The selection of differentially expressed protein spots for MS/MS analysis was based on fold change >2.0 in abundance (Table S1), with a consistent change in the replicate gels of the three biological replicates.

### Mass spectrometry and protein identification

Protein spots with abundance fold change >2.0 between male and female were excised and subjected to in-gel trypsin digestion [16]. Picked gel plugs were extensively washed with 50% (v/v) acetonitrile and totally dried out with 100% acetonitrile. Then, proteins were reduced (10 mM DTT), alkylated (50 mM iodoacetic acid) and then digested with sequencing-grade modified trypsin (Promega, Madison, WI, USA) in 50 mM ammonium bicarbonate for 16 h at 37°C. The digested peptides were extracted with 5% formic acid in 100% acetonitrile (1∶1) and analyzed on a MALDI-TOF/TOF analyzer (ultraflex III, Bruker, Germany).

MS/MS spectra were acquired in the positive-ion mode and automatically submitted to Mascot 2.2 (http://www.matrixscience.com, Matrix Science) for peptide mass finger printings against the NCBInr 20120922 database (20,543,454 sequences, http://www.ncbi.nlm.nih.gov/). The taxonomy was green plants (1093036 sequences). The search parameters were as follows: type of search: MS/MS ion search; enzyme: trypsin; fixed modifications: carbamidomethyl (C); variable modifications: acetyl (protein N-terminal), oxidation (M); mass values: monoisotopic; protein mass: unrestricted; peptide mass tolerance: ±50 ppm; fragment mass tolerance: ±0.2 Da; max missed cleavages: 1; instrument type: MALDI-TOF-TOF. Only significant scores defined by Mascot probability analysis greater than “identity” were considered for assigning protein identity (Figure S2). All of the positive protein identification scores were significant (P<0.05, score>60). The function of the identified proteins was determined based on their ontologies in UniProtKB (http://www.uniprot.org/uniprot/) and previous studies on their homologues. Protein subcellular localization was based on the annotation of the identified proteins in UniProtKB and the prediction by Plant-mPLoc (http://www.csbio.sjtu.edu.cn/bioinf/plant-multi/) and CELLO (http://cello.life.nctu.edu.tw/).

## Results

### Protein extraction by phenol-based protocol

To identify sex-associated proteins in *P. chinensis*, leaves and stems from male and female plants were used for comparative proteomic analysis. In preliminary 2-DE experiments, only a small number of protein spots were observed to exist in the highly acidic and basic area when pH 3–10 Immobiline gels were used (data not shown). Therefore, in order to improve protein resolution, the pH range of IEF gel was reduced from 3–10 to 4–7 for further 2-DE.

**Figure 1:**
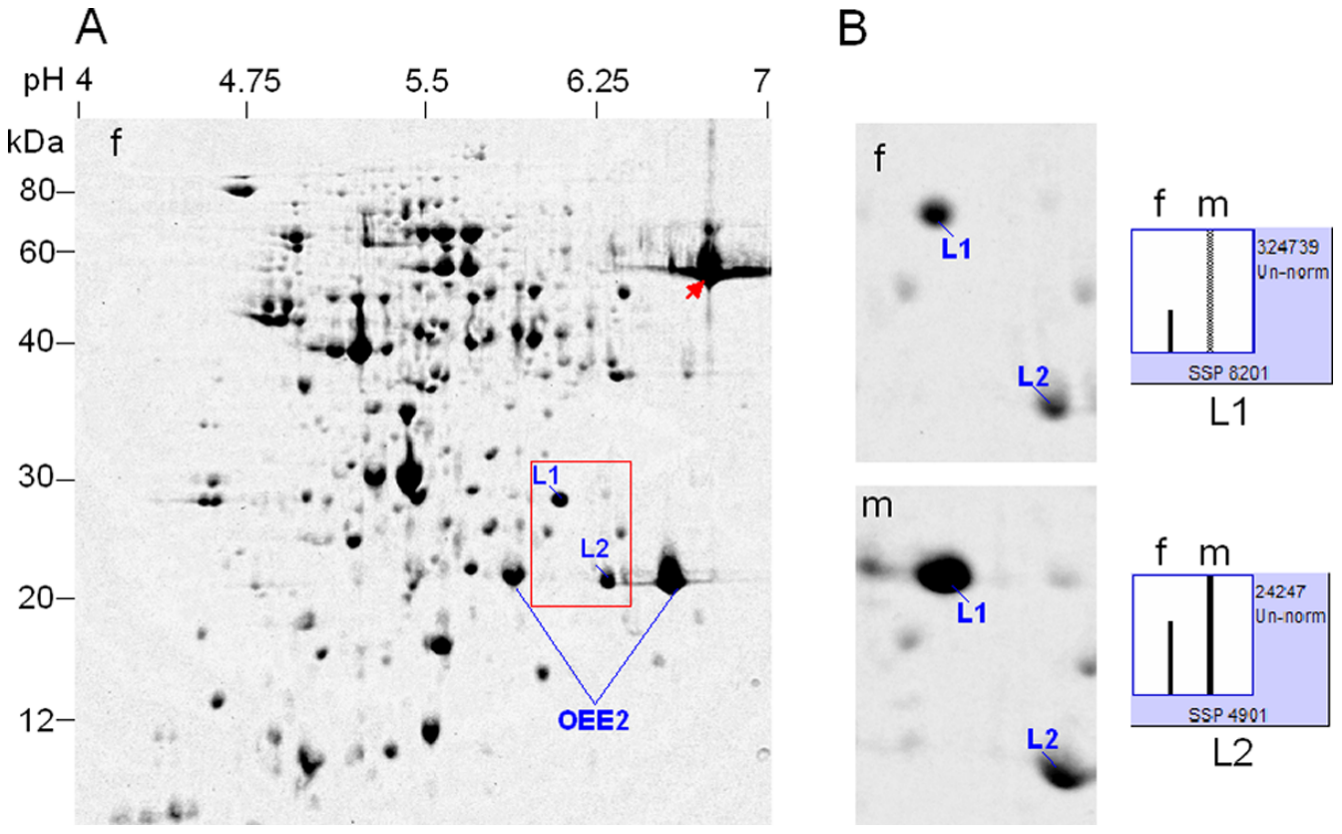
2-DE identification of differentially expressed proteins in leaves between male and female plants (10-year-old) in *Pistacia chinensis*. A mixed tissue powder from three different male or female individuals was used for protein extraction. **A**, 2-DE profile of leaf proteins from female plants as reference. **B**, magnified gel regions containing spots L1 and L2, accompanied by column configuration of relative abundance (generated by PDQUEST). f = female; m = male. OEE2 = oxygen-evolving enhancer protein 2. Arrow indicates the prominent spot Rubisco.

**Figure 2:**
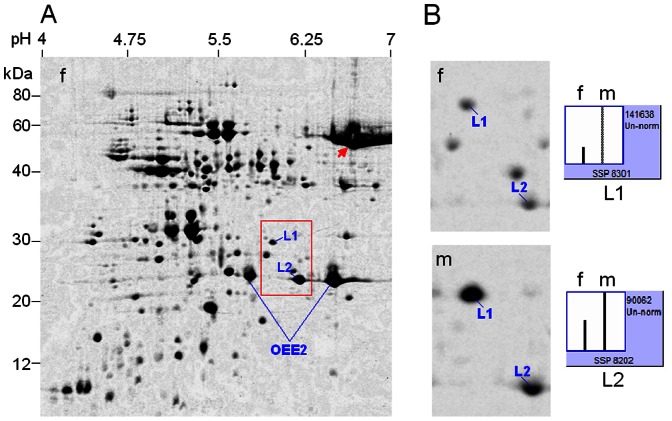
2-DE identification of differentially expressed proteins in leaves between male and female plants (40-year-old) in *Pistacia chinensis*. A mixed tissue powder from three different male or female individuals was used for protein extraction. **A**, 2-DE profile of leaf proteins from female plants as reference. **B**, magnified gel regions containing spots L1 and L2, accompanied by column configuration of relative abundance (generated by PDQUEST). f = female; m = male. OEE2 = oxygen-evolving enhancer protein 2. Arrow indicates the prominent spot Rubisco.

**Figure 3:**
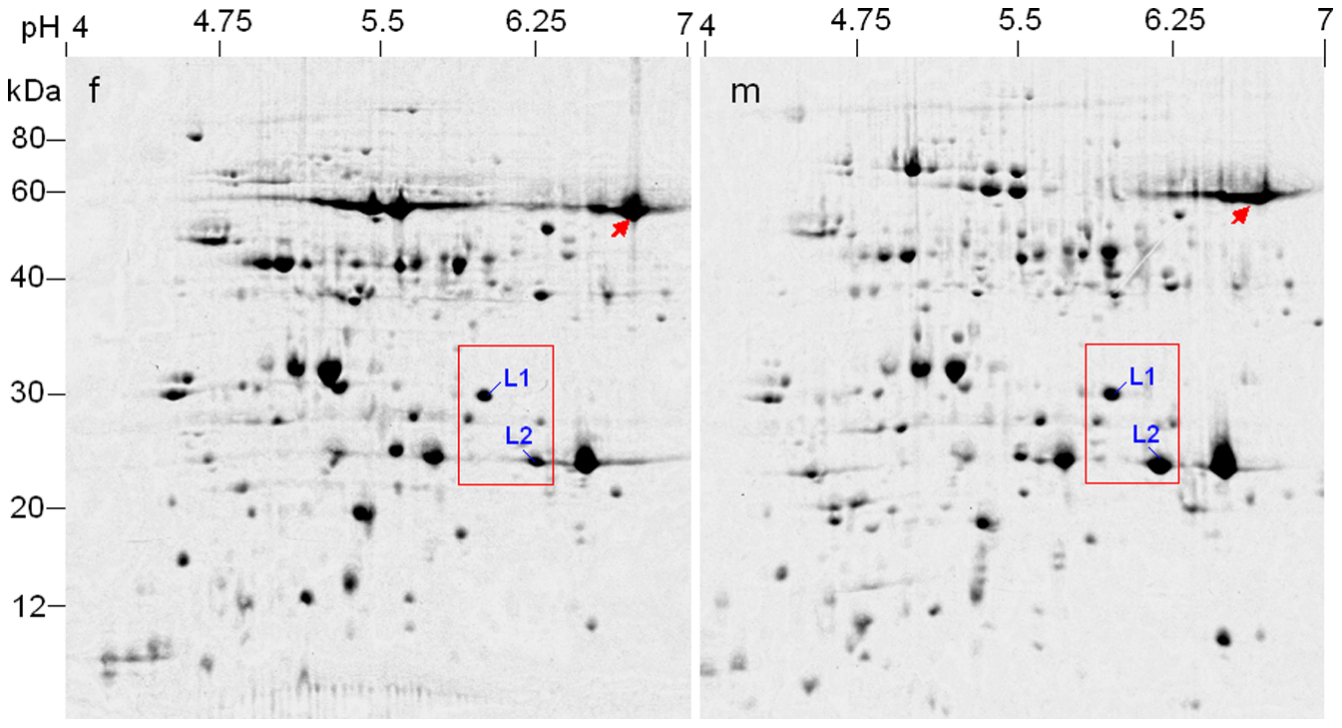
2-DE identification of differentially expressed proteins in leaves between male and female individuals (10-year-old) in *Pistacia chinensis*. Two representative 2-DE gels from three biological replicas are shown. f = female; m = male. OEE2 = oxygen-evolving enhancer protein 2. Arrow indicates the prominent spot Rubisco.

**Figure 4:**
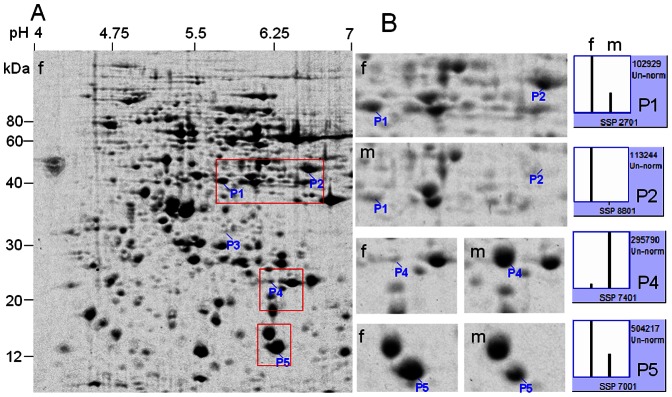
2-DE analysis of differentially expressed proteins in stem phloems between male and female plants (10-year-old) in *Pistacia chinensis*. Stem was sampled in winter. A mixed tissue powder from three different male or female individuals was used for protein extraction. **A**, 2-DE profile of phloem proteins from female plants as reference. **B**, magnified gel regions containing spots P1, P2, P4 and P5, accompanied by column configuration of relative abundance (generated by PDQUEST). Spot P3 failed to be identified by MS/MS. f = female; m = male.

**Figure 5:**
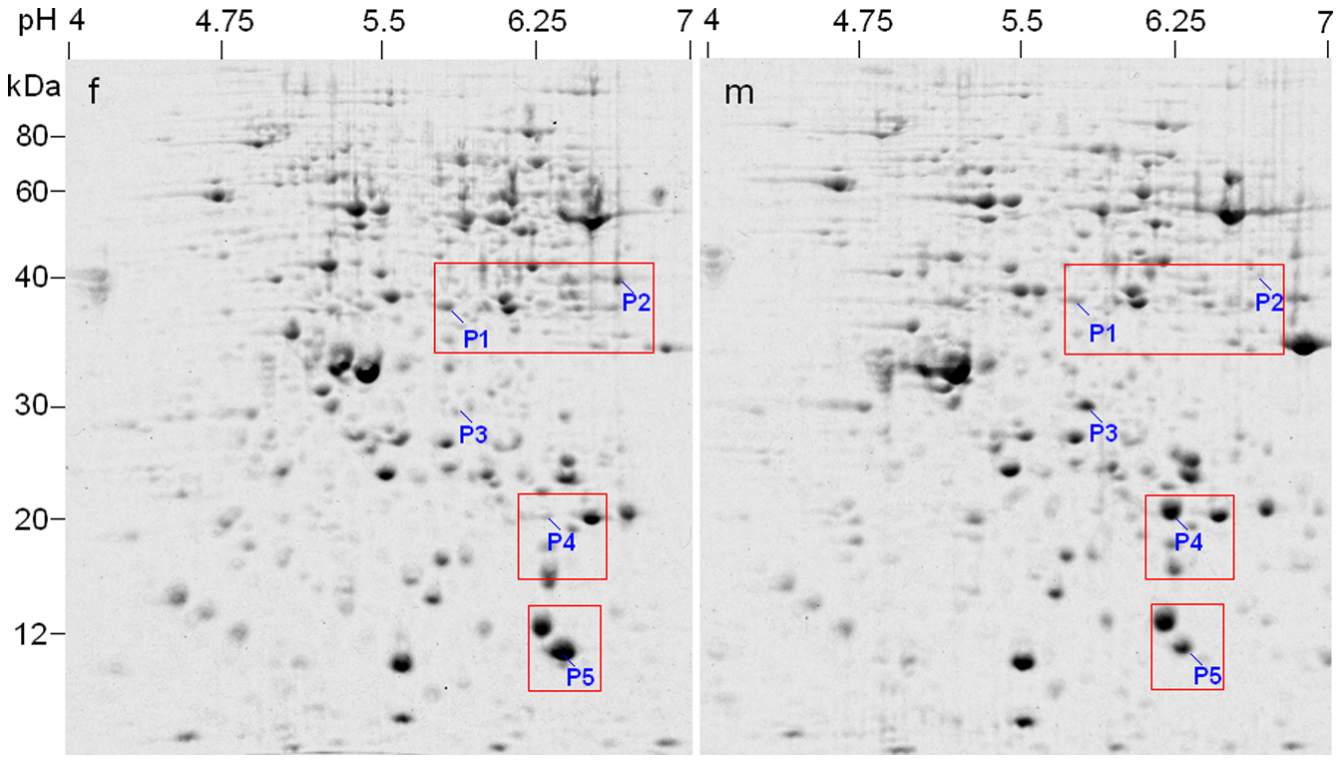
2-DE identification of differentially expressed proteins in stem phloem between male and female individuals (10-year-old) in *Pistacia chinensis*. Two representative 2-DE gels from three biological replicas are shown. Stem was sampled in winter. f = female; m = male.

**Figure 6:**
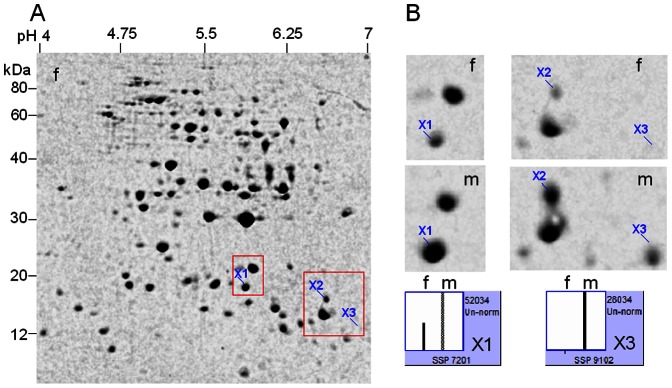
2-DE identification of differentially expressed proteins in stem xylems between male and female plants (10-year-old) in *Pistacia chinensis*. Stem was sampled in winter. A mixed tissue powder from three different male or female individuals was used for protein extraction. **A**, 2-DE profile of xylem proteins from female plants as reference. **B**, magnified gel regions containing spots X1-X3, accompanied by column configuration of relative abundance (generated by PDQUEST). Spot X2 failed to be identified by MS/MS. f = female; m = male.

**Figure 7:**
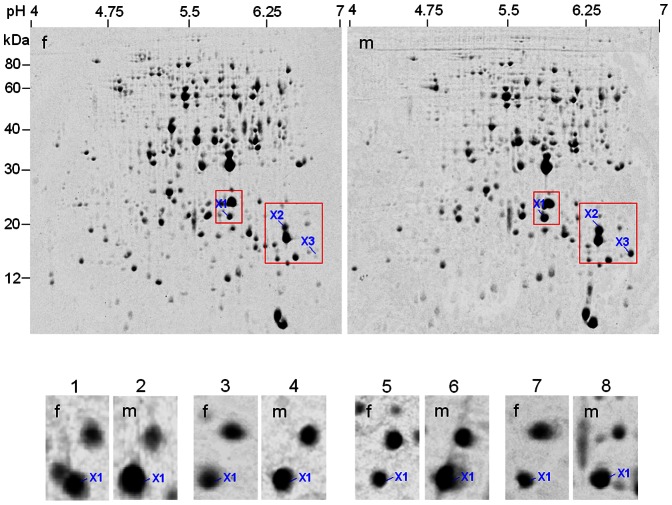
2-DE identification of differentially expressed proteins in stem xylem between male and female individuals (10-year-old) in *Pistacia chinensis*. *Up panel*, two representative CBB-stained gels, xylem was sampled in winter; *down panel*, magnified gel regions containing spots X1 (APX) out of a total of 8 individuals, of which 1–4 were sampled in winter and 5–8 sampled in autumn. f = female; m = male.

The phenol-based protocol can effectively extract protein from tissue power of *P. chinensis*, with a protein yield 5.4, 6.35 and 1.65 mg/g fresh weight for leaf, stem phloem and stem xylem, respectively. By this protocol, leaf protein yield was comparable to those of other woody species [15]; especially xylem protein yield was much higher than that of poplar xylem by 20 mM Tris extraction [17]. More importantly, the phenol-based protocol was able to produce well-resolved and reproducible 2-DE protein profiles (Figure 1–7). In general, approximately 360±8, 492±10 and 193±5 CBB-stained spots (Mr 10 to 80 kDa, pI 4.5 to 7.0) in 2-DE gels (protein load 500 µg) were reproducibly detected in leaf, stem phloem and stem xylem extracts, respectively. Relatively less protein spots were detected in the xylem, in which metabolic activities are weak and the majority of cells are dead.

### Proteomic analysis of differentially expressed proteins in leaves

The protein profiles of leaves (average sample) between male and female 10-year-old plants of *P. chinensis* were compared by 2-DE (Figure 1). PDQUEST analysis indicated that 98.6% of total protein spots were matched, unchanged in abundance between male and female plants across all the gels (Figure S3). On the other hand, spot-to-spot comparison revealed that two leaf protein spots (L1 and L2) were significantly more abundant in male plants than in female plants, especially spot L1 having a 3 fold abundance change.

As such, the significant abundance differences of spots L1 and L2 were also observed in leaves of 40-year-old plants, between male and female (Figure 2, Figure S4). Obviously, the differences in protein profile between sexes may be independent of tree age, because the ages of leaves from 10- and 40-year-old were almost the same.

The abundance differences of spots L1 and L2 between male and female plants were obtained using protein samples from individuals (Figure 3), although the protein profiles of individuals were somewhat different to those of average samples, especially the prominent spot Rubisco (indicated by arrow), characteristic of leaf extract.

MS/MS analysis and homogeneous sequence research showed the two spots were all chloroplast proteins, representing NB-ARC domain containing protein (spot L1) and light harvesting chlorophyll a/b binding protein (LHCII, spot L2), respectively (Table 1). In addition, two prominent, ‘landmark’ spots were identified by MS/MS as the isoforms of oxygen-evolving enhancer protein 2 (OEE2) in chloroplasts.

**Table 1:** Differentially expressed proteins identified by MS/MS analysis in vegetative tissues between male and female plants in *Pistacia chinensis*.

Spot	Relative abundance ratio (female/male)	Identified protein (species)	UniProtKB accession	pI/Mr	Mascot Score	Coverage	Matched peptides	Organ/tissue	Subcellular localization	Molecular function
**Protein spots whose abundance significantly decreased in the female plants in ** ***P. chinensis***										
L1	28.9/100	NB-ARC domain containing protein [*Oryza sativa*]	Q2QNU4	8.17/94.1	76	25%	NHLNHLDK; TNHQIHGVIK; KPSSFKSHK; VLFPMSFAKLR; SHKVLFPMSFAK(2); IVFSSEKTFELK; LAVQDAKLGSK; VKLAQGLMLGDMK; DIMDQVKK; SYASPEAREQLSK; MYSHIDETWETK; ELSYDIEDAIDTFMLK; NILLQLDEK; TCLLSLSKYTEDELIR; VLFPMSFAK(2); EQLSKVR; SLIQPMSENTLWDEDGK(2); VNKSISLGLENNLDVDNMR; IIVTTRNK; VLQLEDCSGMDKNHLNHLDK	Leaf	Chloroplast^a^	ADP binding
L2	52/100	Light harvesting chlorophyll a/b-binding protein (LHCII) [*Arabidopsis thaliana*]	Q9SY97	8.61/29.2	69	4%	WLAYGEIINGR	Leaf	Chloroplast^a^	Light harvesting
P4	6.7/100	Temperature-induce lipocalin (TIL) [*Solanum lycopersicum*]	Q38JE1	5.96/21.3	73	30%	NLDVEKYMGR; YMGRWYEIASFPSR; KYLWILCR; YLWILCR; GFWWIK; ATYTLNQDGTVHVLNETWSGGKR	Stem phloem	Cytoplasm^b^	Transporter activity
X1	47.2/100	Asorbate peroxidase (APX) [*Pennisetum americanum*]	A4ZYP9	5.69/27.5	88	24%	SCAPLMLR; EDKPQPPPEGR; QMGLSDQDIVALSGGHTLGRCHK; TLLSDPVFRPLVEKYAADEK	Stem xylem	Cytoplasm^b^	Peroxidase activity Heme binding
X3	2.9/100	Temperature-induced lipocalin (TIL) [*Ricinus communis*]	B9SBY6	6.33/21.6	143	7%	THLDDEIYNQLVEK	Stem xylem	Cytoplasm^b^	Transporter activity
**Protein spots whose abundance significantly increased in the female plants in ** ***P. chinensis***										
P1	100/34.2	Eukaryotic translation initiation factor 5A2 ( eIF-5A2) [*Tamarix androssowii*]	H9BNZ5	5.60/17.6	67	55%	SDEEHHFESKADAGASK; LPTDDTLLTQIK; NRPCKVVEVSTSK; TDYQLIDISEDGFVSLLTETGGTKDDLR; DLIVSVMSSMGEEQICGLK	Stem phloem	Cytoplasm^b^	Initiation factor
P2	100/-	Phosphoglycerate kinase 2 (PGK) [*Vitis vinifera*]	A5CAF8	8.26/50.2	121	4%	LVTALPDGGVLLLENVR	Stem phloem	Cytoplasm^a^	Kinase Transferase
P5	100/40.0	Expressed protein [*Populus trichocarpa*]	B9PDT9	6.34/17.2	74	33%	GLTNLETLRLR; ASLQILRHASPVNVEK; SLFPLAMASGLPNLQILR;	Stem phloem	Cytoplasm^b^; PM^b^	Unclear

**Note:** -, undetected by PDQUEST analysis. Spot P3 in Figure 3 and spot X2 in Figure 4 failed to be identified by MS/MS and are not listed in the table.

a , subcellular l in UniProtKB;

b , subcellular localization predicted by software.

PM = Plasma membrane.

### Proteomic analysis of differentially expressed proteins in stems

The stem protein profiles between male and female plants of *P. chinensis* were compared by 2-DE. Stem xylem and stem phloem were separated from twigs (1–2 year-old) of 10-year-old plants.

In protein extract of stem phloem (average sample), 5 spots were found to be differentially expressed between male and female, of which 2 were less abundance and the other 3 spots were more abundance in female plants (Figure 4, Figure S5). Four spots were successfully identified by MS/MS and represented distinct proteins in NCBI or UniProtKB protein databases (Table 1). Most obviously, temperature-induced lipocalin (TIL, spot P4) existed in 14-fold higher abundance in the male than in the female, while eukaryotic translation initiation factor 5A2 (eIF-5A2, spot P1), phosphoglycerate kinase 2 (PGK, spot P2) and an expressed protein (spot P5) existed in higher abundance in the female. The abundance differences between male and female plants mentioned above were confirmed using protein samples from individuals (Figure 5).

In the protein extract of stem xylem (average sample), 3 spots were significantly changed in abundance between male and female plants (Figure 6, Figure S6), of which 2 were identified by MS/MS. Asorbate peroxidase (APX, spot X1) and TIL (spot X3) were more abundant in the male (Table 1). We were very interest in xylem APX, because its absolute abundance is higher (than TIL) in male plants and the abundance difference is sharp between both sexes.

In 10 individuals examined, the abundance difference of xylem APX between both sexes was very significant (Figure 7), as found using average samples. Moreover, xylems sampled from autumn and winter had similar difference (Figure 7).

## Discussion

In dioecious plants cultivated for fruit or seed such as *P. chinensis*, it is difficult to identify females at juvenile stage, particularly in the absence of well defined sex chromosomes. Females in dioecious plant populations often invest more in reproduction and less in growth and maintenance than males [18]. This differential investment between sexes may result in distinct growth patterns [19] and sex-specific responses to environmental stresses [20]. It is expected that such difference would display at the proteome level. Therefore, vegetative tissues, rather than reproductive tissues were used to find out proteins whose abundance changes significantly between both sexes, which will provide a cue for sex identification in *P. chinensis* prior to flowering. Especially, xylem would be a good choice as material for sex determination, since xylem exists inside the stem, less affected by the external conditions than leaf and stem phloem, more easily separated out from the stem than phloem all the year around.

The phenol-based protein extraction protocol has been proved to work well in various plant tissues, including woody tissues [14], [15]. This protocol is also suitable for *P. chinensis*. Protein yields are high. The 2-DE analysis reveals well-resolved of most protein spots throughout the gel with little streaking, indicating the high quality of the protein preparation.

In this study, 2-DE analysis has revealed a total of 10 differential protein spots (p≤0.05) in vegetative tissues between male and female plants in *P. chinensis*. As the *P. chinensis* genome has not yet been sequenced, MS/MS analysis followed by database searches of proteins in the taxonomy of green plants has been used to identify proteins of interest, as in other unsequenced plant species [21]. As a result, 7 spots had a significant match (MASCOT score>69; Table 1) with 6 functional proteins from *Oryza sativa* (rice), *Arabidopsis thaliana* (mouse ear cress), *Pennisetum americanum* (pearl millet), *Ricinus communis* (castor oil plant), *Solanum lycopersicum* (tomato), *Tamarix androssowii* (salt cedar) and *Vitis vinifera* (grape vine). In particular, PGK existed in high abundance in the stem phloem in female, but almost not detected in the male. TIL was highly abundant in stem xylem and stem phloem in male plants, while its expression was much low in female plants. Blast analysis showed that TIL (Q38JE1) in the stem phloem and that (B9SBY6) in the stem xylem share high identities (85%) and positives (94%). Hence, it is assumed that PGK and TIL might be promising candidates to serve as molecular markers for sex determination in *P. chinensis*. In addition, these differentially expressed proteins between both sexes in *P. chinensis* are highly tissue specific: two in chloroplasts of leaves, two in stem xylem and three in stem phloem. These differences in protein expression found in adult plants are needed to be examined in juvenile plants of *P. chinensis*. Obviously, protein profiles of plant tissues of the same type are similar and undependable of tree age. This is reasonable because the actual ages of leaves or twigs are similar, no matter from 10- or 40-year-old plants.

In this study, most of the identified proteins including NB-ARC domain containing proteins, LHCII, APX, TIL, eIF-5A2 and PGK are involved in defence and stress responses (Table 1). Most resistance proteins have a centrally located NB-ARC domain, which consists of three subdomains: a nucleotide-binding (NB) domain and a C-terminal extension that forms a four-helix bundle (ARC1) and a winged-helix fold (ARC2) [22]. Mutations in the NB-ARC domain often abolish R-protein function. The NB-ARC domains for tomato (*Lycopersicon esculentum*) R-proteins are functional ATPase modules that specifically bind and hydrolyze ATP *in* vitro [23]. LHCII is the important component of the light harvesting complex [24], involved in light reactions in photosynthesis. More LHCII in male *P. chinensis* is consistent with a previous observation that male plants show higher chlorophyll a/b ratio than female plants [25]. APX is well known to play an essential role in scavenging reactive oxygen species, with ascorbate serving as the electron donor [26]. TIL is involved in modulating plant tolerance to oxidative and temperature stresses [27]–[29]. Arabidopsis eIF-5A2 plays a crucial role in plant growth and development by regulating cell division, cell growth and cell death [30]. Cytoplasmic PGK, catalyzing the formation of ATP to ADP and *vice versa*, takes part in glycolysis in plants [31]. PGK levels were enhanced by low temperature stress in rice leaf blades [32] and by salt stress in rice leaf laminas [33], suggesting that it is an early responsive protein to both salt stress and cold stress. Furthermore, the identified sex-related proteins in *P. chinensis* are stress/defence proteins, and exist in more abundance in male than in female, suggesting an increased resistance of male plants. This agrees with previous studies in several dioecious plants in response to salinity, drought stress [34], [35] and other environmental stresses [36], and females usually show a lower tolerance capacity compared with males.

In addition, the identified proteins related to sex in *P. chinensis* here are all functional proteins. Previous studies have showed that there existed various functional, housekeeping gene differences between male and female in dioecious plants. For instance, it is suggested that *Arabidopsis thaliana* homologs of shootmeristemless (STM) and cup shaped cotyledon 1 (CUC1) and CUC2 genes in *S. latifolia* could be strong candidates in the involvement of sex determination [37]. In dioecious Kiwifruit (*Actinidia chinensis*), 15 differentially expressed genes have been isolated and sequenced from male and female floral buds, including pectin methylesterase (a cell-wall modifying enzyme) and a ADP-ribosylation factor (a small GTP-binding protein) [38], both of which are abundantly expressed in male floral buds than in female floral buds. It is suggested that sex determining genes may be present in the genomes of hermaphroditic plants, but may have quite different functions in some plants [3].

In addition, a range of sex-linked molecular markers such as RAPD, RFLP, AFLP and microsatellite have been generated for sex determination in dioecious plants. These techniques need to design and try different sets of random primers [4]–[7] and the function of amplified products is often unclear [5], [6], while proteomic analysis directly identify the changes of gene products (proteins) in abundance [10] and in most cases the functions of these proteins identified are known. As for sex determination, both techniques are appropriate if they can distinguish male and female plants.

In conclusion, our study has revealed that several functional, stress proteins change in abundance greatly between both sexes in *P. chinensis*. The identified sex-related proteins will be useful to better understand the molecular events of sex differentiation. Three proteins (APX, PGK and TIL) might serve as molecular markers for sex determination in *P. chinensis*. The results form the basis for a further understanding of the biochemical mechanisms of sex determination in *P. chinensis*.

## Supporting Information

Figure S1 **The appearance difference between male and female flowers in** ***P. chinensis*** **.** Male panicles are pale green (**A**) and female panicles are purple (**B**).
(TIF)

Figure S2 **Plant materials from** ***Pistacia chinensis*** **used for proteomic analysis.** **A**, compound leaves. **B**, a segment of twig, separated stem phloem and separated stem xylem. No discernible differences in appearance were observed in leaves or twigs between male and female.
(ZIP)

Figure S3 **2-DE comparison of leaf protein profiles between male and female plants (10-year-old).** **A, B**, representative 2-DE maps (protein load 500 µg, CBB stained) from two independent experiments. OEE2 = oxygen-evolving enhancer protein 2.
(TIF)

Figure S4 **2-DE comparison of leaf protein profiles between male and female plants (40-year-old).** **A, B**, representative 2-DE maps (protein load 500 µg, CBB stained) from two independent experiments. OEE2 = oxygen-evolving enhancer protein 2.
(TIF)

Figure S5 **2-DE comparison of stem phloem leaf protein profiles between male and female plants (10-year-old).** **A, B**, representative 2-DE maps (protein load 500 µg, CBB stained) from two independent experiments.
(TIF)

Figure S6 **2-DE comparison of stem xylem leaf protein profiles between male and female plants (10-year-old).** **A, B**, representative 2-DE maps (protein load 500 µg, CBB stained) from two independent experiments.
(TIF)

Table S1 **The normalized volume and relative ratio of differentially expressed protein spots between the male samples and female samples.** The data were the mean from three independent experiments. The differences in abundance between both sexes were significant by one-way ANOVA (p<0.05). f = female; m = male.
(DOC)
