# Supplementary figures and images for: Proteomic Identification of Differentially Expressed Proteins between Male and Female Plants in Pistacia chinensis
Source: PLoS One. 2013 May 17;8(5):e64276. doi: 10.1371/journal.pone.0064276 (PMC3656840; doi:10.1371/journal.pone.0064276)

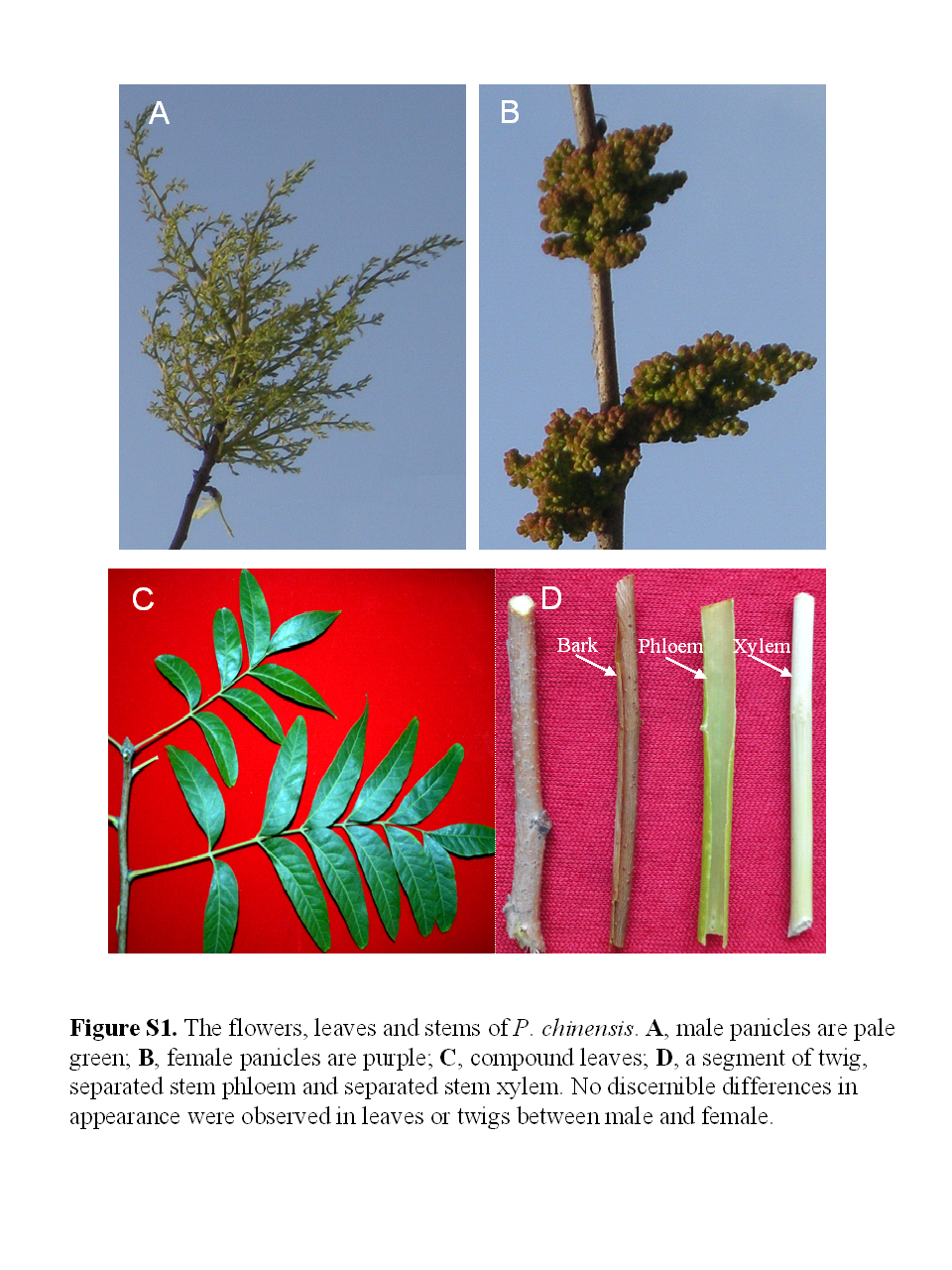

Supplement: Figure S1 — The appearance difference between male and female flowers in P. chinensis . Male panicles are pale green (A) and female panicles are purple (B). (TIF) [file pone.0064276.s001.tif]

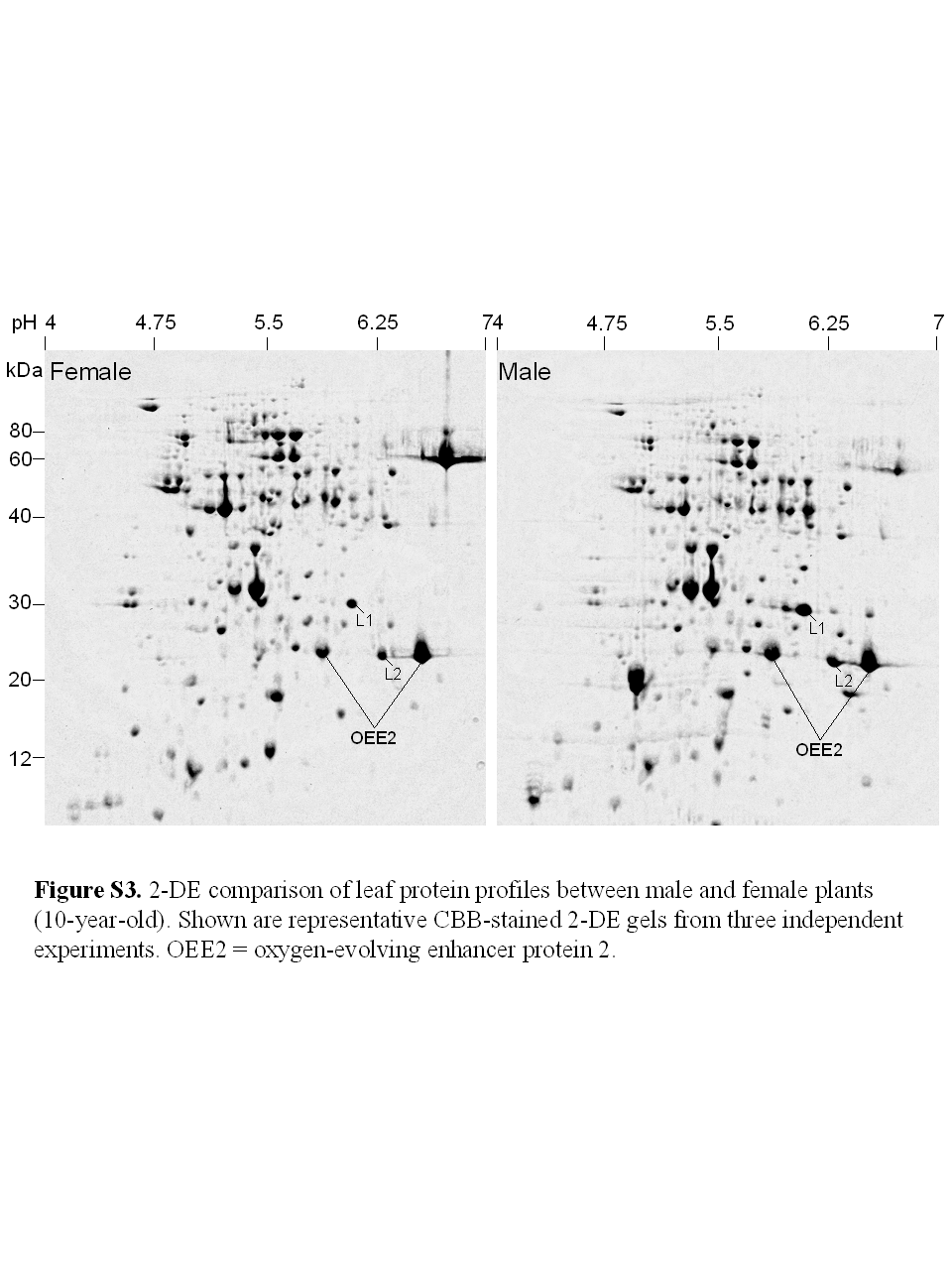

Supplement: Figure S3 — 2-DE comparison of leaf protein profiles between male and female plants (10-year-old). A, B, representative 2-DE maps (protein load 500 µg, CBB stained) from two independent experiments. OEE2 = oxygen-evolving enhancer protein 2. (TIF) [file pone.0064276.s003.tif]

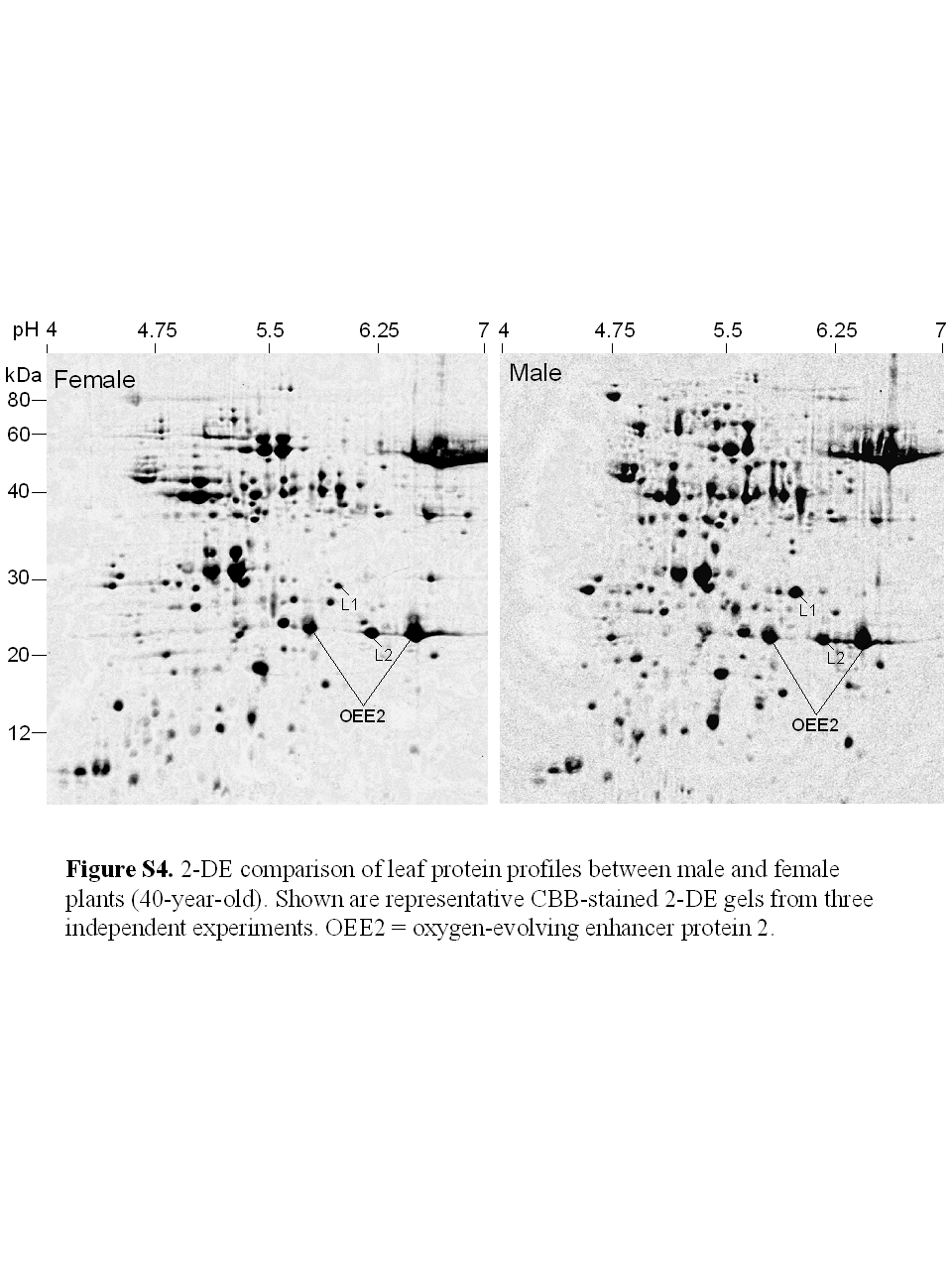

Supplement: Figure S4 — 2-DE comparison of leaf protein profiles between male and female plants (40-year-old). A, B, representative 2-DE maps (protein load 500 µg, CBB stained) from two independent experiments. OEE2 = oxygen-evolving enhancer protein 2. (TIF) [file pone.0064276.s004.tif]

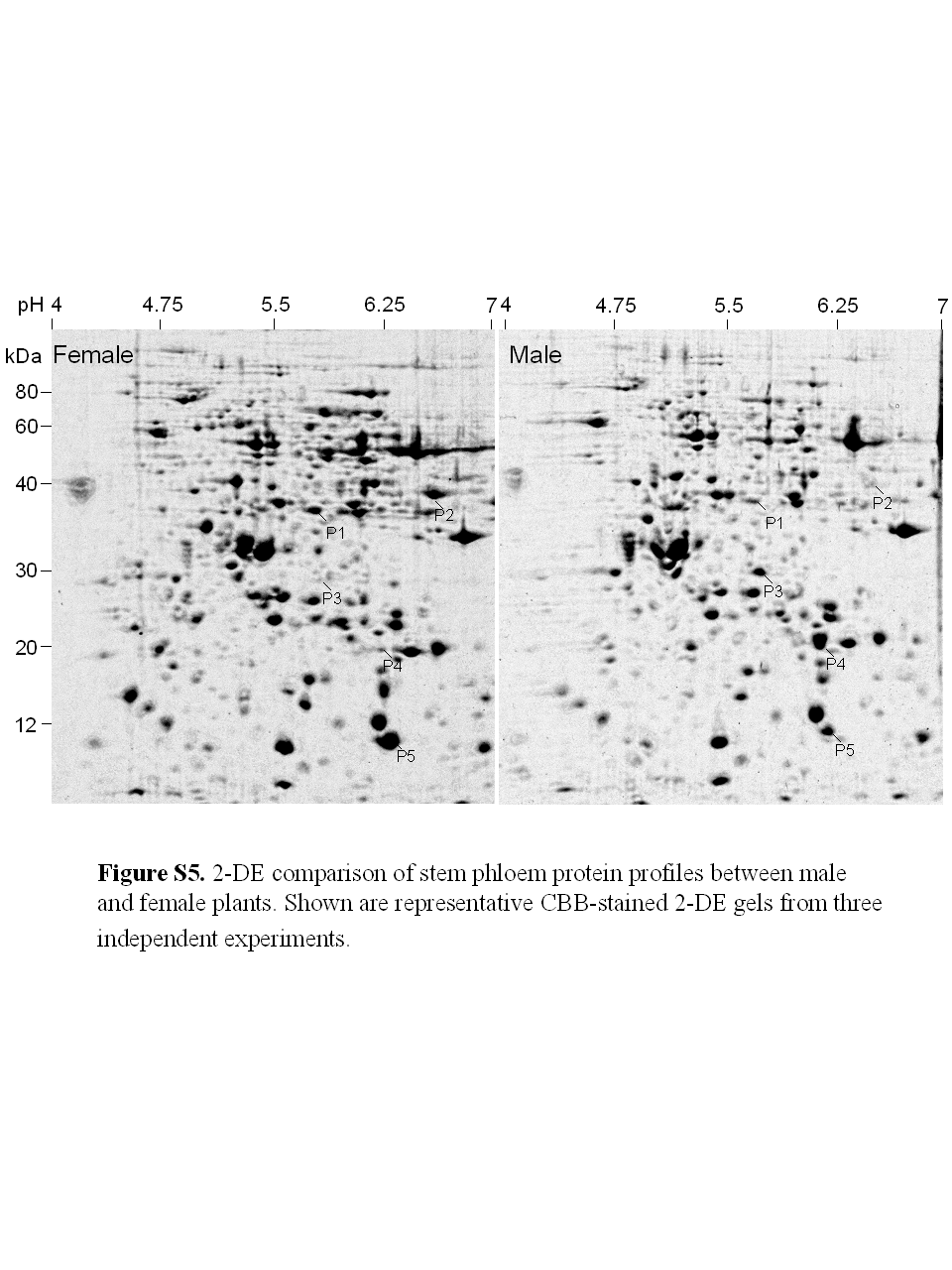

Supplement: Figure S5 — 2-DE comparison of stem phloem leaf protein profiles between male and female plants (10-year-old). A, B, representative 2-DE maps (protein load 500 µg, CBB stained) from two independent experiments. (TIF) [file pone.0064276.s005.tif]

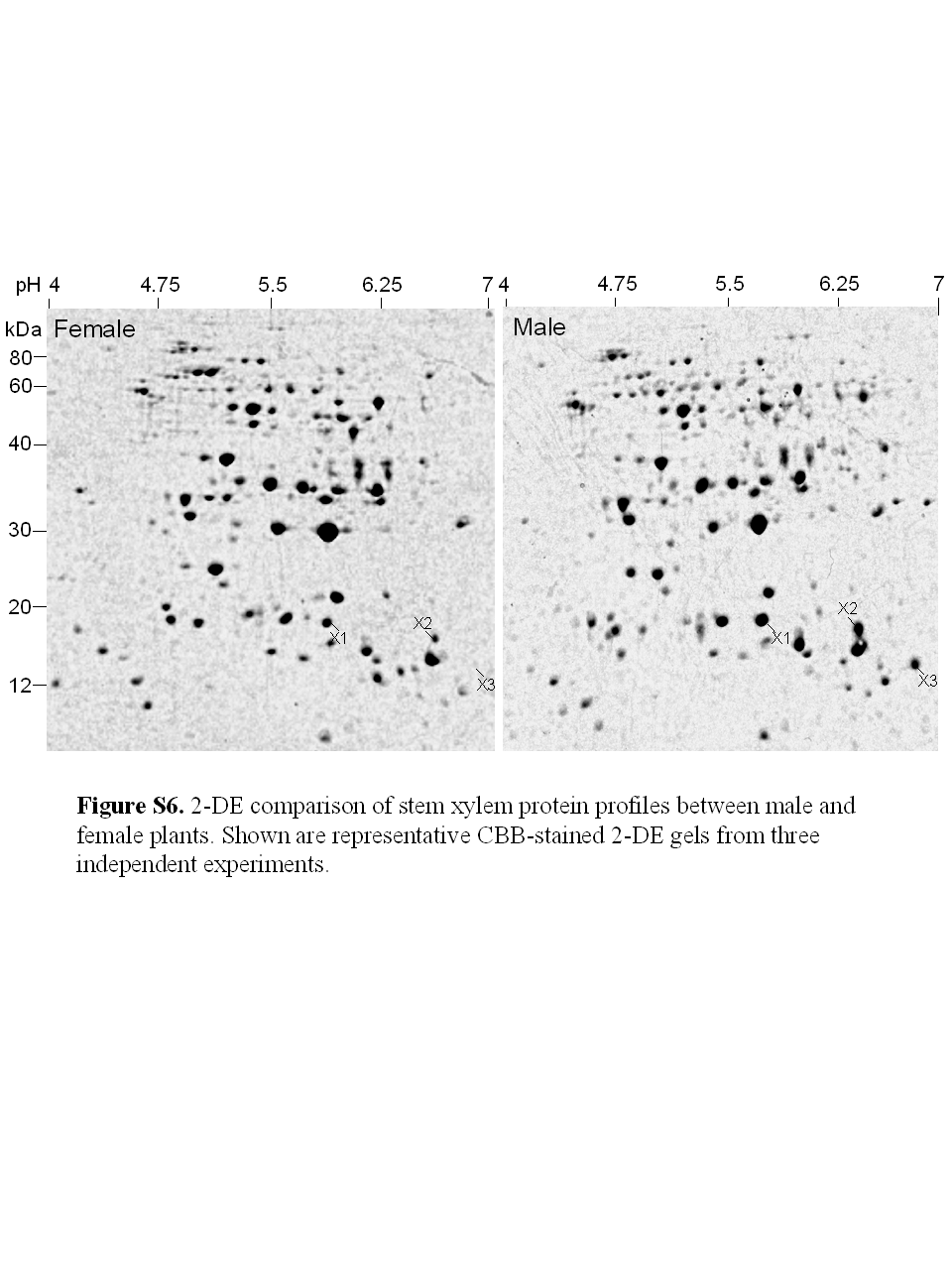

Supplement: Figure S6 — 2-DE comparison of stem xylem leaf protein profiles between male and female plants (10-year-old). A, B, representative 2-DE maps (protein load 500 µg, CBB stained) from two independent experiments. (TIF) [file pone.0064276.s006.tif]
