# Supplementary material for: Proteomic Identification of Differentially Expressed Proteins between Male and Female Plants in Pistacia chinensis
Source: PLoS One. 2013 May 17;8(5):e64276. doi: 10.1371/journal.pone.0064276 (PMC3656840; doi:10.1371/journal.pone.0064276)
Supplement: Figure S2 — Plant materials from Pistacia chinensis used for proteomic analysis. A, compound leaves. B, a segment of twig, separated stem phloem and separated stem xylem. No discernible differences in appearance were observed in leaves or twigs between male and female. (ZIP) [file pone.0064276.s002.zip › Figure S2/spot L2/L2ú¿G12).PDF]

Protein Group

RecName: Full=Protein FAM198A; Flags: Precursor	gi|123795368
hypothetical protein LOC245050 isoform 2 [Mus musculus]	gi|315623947

Peptide Information

Calc. Mass	Obsrv. Mass	± da	± ppm	Start Seq.	End Seq.	Sequence	Ion Score	C. I. %	Modification	Rank	Result Type
1801.9569	1801.9556	-0.0013	-1	54	69	QPARNLGHWTGQALPR	25	0		2	Mascot

Gel Idx/Pos	166/G12	Instr./Gel Origin	ab347000171/20110905	Process Status	Analysis Succeeded
Plate [#] Name	[1] wjl	Instrument Sample Name		Spectra	6

Rank	Protein Name	Accession No.	Total Ion Score	Total Ion C. I. %
1	light-harvesting complex I chlorophyll a/b binding protein 3 [Arabidopsis thaliana]	gi|15219941	69	99.974

Peptide Information

Calc. Mass	Obsrv. Mass	± da	± ppm	Start Seq.	End Seq.	Sequence	Ion Score	C. I. %	Modification	Rank	Result Type
1291.6793	1291.7227	0.0434	34	95	105	WLAYGEIINGR	69	99.974		1	Mascot

Rank	Protein Name	Accession No.	Total Ion Score	Total Ion C. I. %
2	m032R [Myxoma virus]	gi|9633668	48	97.105

Peptide Information

Calc. Mass	Obsrv. Mass	± da	± ppm	Start Seq.	End Seq.	Sequence	Ion Score	C. I. %	Modification	Rank	Result Type
1031.5924	1031.5529	-0.0395	-38	317	324	KYFIGLYK	48	97.105		1	Mascot

Rank	Protein Name	Accession No.	Total Ion Score	Total Ion C. I. %
3	hypothetical protein [Bacillus cereus]	gi|71067028	44	91.297

Peptide Information

Calc. Mass	Obsrv. Mass	± da	± ppm	Start Seq.	End Seq.	Sequence	Ion Score	C. I. %	Modification	Rank	Result Type
1031.4866	1031.5529	0.0663	64	141	148	QFMEGIYK	44	91.297	Oxidation (M)[3]	2	Mascot

Rank	Protein Name	Accession No.	Total Ion Score	Total Ion C. I. %
4	hypothetical protein [Fusobacterium nucleatum subsp. vincentii ATCC 49256]	gi|34763417	37	61.48

Peptide Information

Calc. Mass	Obsrv. Mass	± da	± ppm	Start Seq.	End Seq.	Sequence	Ion Score	C. I. %	Modification	Rank	Result Type

1031.556	1031.5529	-0.0031	-3	27	34	KYGIFFEK	37	61.48		3	Mascot

5 hypothetical protein AM1037 [Anaplasma marginale str. St. Maries] gi|56417094 37 60.583

Peptide Information

Calc. Mass	Obsrv. Mass	± da	± ppm	Start Seq.	End Seq.	Sequence	Ion Score	C. I. %	Modification	Rank	Result Type
1291.7117	1291.7227	0.011	9	425	436	VSIADYARLAGR	37	60.583		2	Mascot

6 trimethylamine-N-oxide reductase TorA [Haemophilus somnus 2336] gi|170718122 37 57.077

Protein Group

COG0243: Anaerobic dehydrogenases, typically selenocysteine-containing [Actinobacillus pleuropneumoniae serovar 1 str. 4074]	gi|32034680
TorA [Pasteurella multocida subsp. multocida str. Pm70]	gi|15603658
trimethylamine-N-oxide reductase TorA [Actinobacillus succinogenes 130Z]	gi|152978574
trimethylamine-N-oxide reductase precursor [Haemophilus somnus 129PT]	gi|113461809

Peptide Information

Calc. Mass	Obsrv. Mass	± da	± ppm	Start Seq.	End Seq.	Sequence	Ion Score	C. I. %	Modification	Rank	Result Type
1031.4768	1031.5529	0.0761	74	655	662	GHPMWFEK	37	57.077		4	Mascot

7 hypothetical protein lpl1800 [Legionella pneumophila str. Lens] gi|54294723 36 53.045

Protein Group

hypothetical protein lpp1799 [Legionella pneumophila str. Paris]	gi|54297748

Peptide Information

Calc. Mass	Obsrv. Mass	± da	± ppm	Start Seq.	End Seq.	Sequence	Ion Score	C. I. %	Modification	Rank	Result Type
1031.5044	1031.5529	0.0485	47	22	29	KYTSEEFK	36	53.045		5	Mascot

8 phospholipid-transporting ATPase [Theileria parva strain Muguga] gi|71031394 35 40.75

Peptide Information

Calc. Mass	Obsrv. Mass	± da	± ppm	Start Seq.	End Seq.	Sequence	Ion Score	C. I. %	Modification	Rank	Result Type
1031.5229	1031.5529	0.03	29	391	398	MKYGLFEK	35	40.75	Oxidation (M)[1]	6	Mascot

9 hypothetical protein DIP1707 [Corynebacterium gi|38234278 35 37.095

diphtheriae NCTC 13129]

Peptide Information

Calc. Mass	Obsrv. Mass	± da	± ppm	Start Seq.	End Seq.	Sequence	Ion Score	C. I. %	Modification	Rank	Result Type
1031.5044	1031.5529	0.0485	47	121	128	KSYTEFEK	35	37.095		7	Mascot

Rank	Protein Name	Accession No.	Total Ion Score	Total Ion C. I. %
10	hypothetical protein BcerKBAB4_0935 [Bacillus weihenstephanensis KBAB4]	gi|163938932	35	35.778

Peptide Information

Calc. Mass	Obsrv. Mass	± da	± ppm	Start Seq.	End Seq.	Sequence	Ion Score	C. I. %	Modification	Rank	Result Type
1031.5231	1031.5529	0.0298	29	2	9	KYMAYVEK	35	35.778		8	Mascot

Gel Idx/Pos	155/G13	Instr./Gel Origin	ab347000171/20110905	Process Status	Analysis Succeeded
Plate [#] Name	[1] wjl	Instrument Sample Name		Spectra	6

Rank	Protein Name	Accession No.	Total Ion Score	Total Ion C. I. %
1	RecName: Full=Oxygen-evolving enhancer protein 2, chloroplastic; Short=OEE2; AltName: Full=23 kDa s	gi|266856	113	100

Peptide Information

Calc. Mass	Obsrv. Mass	± da	± ppm	Start Seq.	End Seq.	Sequence	Ion Score	C. I. %	Modification	Rank	Result Type
1189.6211	1189.6445	0.0234	20	111	120	EVEYPGQVLR	66	99.956		1	Mascot
1801.9231	1801.9572	0.0341	19	106	120	WNPSKEVEYPGQVLR	46	95.834		1	Mascot

Rank	Protein Name	Accession No.	Total Ion Score	Total Ion C. I. %
2	flagellar motor switch protein [Azotobacter vinelandii DJ]	gi|226944511	32	0

Peptide Information

Calc. Mass	Obsrv. Mass	± da	± ppm	Start Seq.	End Seq.	Sequence	Ion Score	C. I. %	Modification	Rank	Result Type
1189.563	1189.6445	0.0815	69	288	297	EDMEARGPIR	32	0	Oxidation (M)[3]	2	Mascot

Rank	Protein Name	Accession No.	Total Ion Score	Total Ion C. I. %
3	hypothetical protein EE36_12843 [Sulfitobacter sp. EE-36]	gi|83943068	27	0

Peptide Information

Calc. Mass	Obsrv. Mass	± da	± ppm	Start Seq.	End Seq.	Sequence	Ion Score	C. I. %	Modification	Rank	Result Type
1189.606	1189.6445	0.0385	32	338	348	EDIDLSTGAIR	27	0		3	Mascot
