# Supplementary material for: Proteomic Identification of Differentially Expressed Proteins between Male and Female Plants in Pistacia chinensis
Source: PLoS One. 2013 May 17;8(5):e64276. doi: 10.1371/journal.pone.0064276 (PMC3656840; doi:10.1371/journal.pone.0064276)
Supplement: Table S1 — The normalized volume and relative ratio of differentially expressed protein spots between the male samples and female samples. The data were the mean from three independent experiments. The differences in abundance between both sexes were significant by one-way ANOVA (p<0.05). f = female; m = male. (DOC) [file pone.0064276.s007.doc]

**Table S1** The normalized volume and relative ratio of differentially expressed protein spots between the male samples and female samples.

| **Spot** | **Volume** (f) | **Volume** (m) | **Relative ratio** (f/m) |
| --- | --- | --- | --- |
| Samples from 10-year-old plants | | | |
| L1 | 39352 | 138638 | 28.4/100 |
| L2 | 47152 | 89671 | 52.6/100 |
| P1 | 110574 | 37275 | 100/33.7 |
| P2 | 115390 | None detected |  |
| P4 | 18717 | 293796 | 6.4/100 |
| P5 | 498637 | 200362 | 100/40.2 |
| X1 | 23284 | 49843 | 46.7/100 |
| X3 | None detected | 30172 |  |
| Samples from 40-year-old plants | | | |
| L1 | 86773 | 295686 | 29.3/100 |
| L2 | 14684 | 28390 | 51.7/100 |

The data were the mean from three independent experiments. The differences in abundance between both sexes were significant by one-way ANOVA (p<0.05). f = female; m = male.
