# Supplementary material for: Proteomic Identification of Differentially Expressed Proteins between Male and Female Plants in Pistacia chinensis
Source: PLoS One. 2013 May 17;8(5):e64276. doi: 10.1371/journal.pone.0064276 (PMC3656840; doi:10.1371/journal.pone.0064276)
Supplement: Figure S2 — Plant materials from Pistacia chinensis used for proteomic analysis. A, compound leaves. B, a segment of twig, separated stem phloem and separated stem xylem. No discernible differences in appearance were observed in leaves or twigs between male and female. (ZIP) [file pone.0064276.s002.zip › Figure S2/spot L2/L2.doc]

G12
